# Peptides with Antimicrobial Activity in the Saliva of the Malaria Vector *Anopheles coluzzii*

**DOI:** 10.3390/ijms25105529

**Published:** 2024-05-18

**Authors:** Giulia Bevivino, Linda Maurizi, Maria Grazia Ammendolia, Catia Longhi, Bruno Arcà, Fabrizio Lombardo

**Affiliations:** 1 Department of Public Health and Infectious Diseases, Sapienza University of Rome, P.le Aldo Moro 5, 00185 Rome, Italy; giulia.bevivino@uniroma1.it (G.B.); linda.maurizi@uniroma1.it (L.M.); catia.longhi@uniroma1.it (C.L.); bruno.arca@uniroma1.it (B.A.); 2 National Center for Innovative Technologies in Public Health, Italian National Institute of Health, Viale Regina Elena 299, 00161 Rome, Italy; maria.ammendolia@iss.it

**Keywords:** mosquito, salivary glands, innate immunity, antimicrobial peptides

## Abstract

Mosquito saliva plays a crucial physiological role in both sugar and blood feeding by helping sugar digestion and exerting antihemostatic functions. During meal acquisition, mosquitoes are exposed to the internalization of external microbes. Since mosquitoes reingest significant amounts of saliva during feeding, we hypothesized that salivary antimicrobial components may participate in the protection of mouthparts, the crop, and the gut by inhibiting bacterial growth. To identify novel potential antimicrobials from mosquito saliva, we selected 11 candidates from *Anopheles coluzzii* salivary transcriptomic datasets and obtained them either using a cell-free transcription/translation expression system or, when feasible, via chemical synthesis. Hyp6.2 and hyp13, which were predicted to be produced as propeptides and cleaved in shorter mature forms, showed the most interesting results in bacterial growth inhibition assays. Hyp6.2 (putative mature form, 35 amino acid residues) significantly inhibited the growth of Gram-positive (*Staphylococcus aureus*) and Gram-negative (*Escherichia coli* and *Serratia marcescens*) bacteria. Hyp13 (short form, 19 amino acid residues) dose-dependently inhibited *E. coli* and *S. marcescens* growth, inducing membrane disruption in both Gram-positive and Gram-negative bacteria as indicated with scanning electron microscopy. In conclusion, we identified two *A. coluzzii* salivary peptides inhibiting Gram-positive and Gram-negative bacteria growth and possibly contributing to the protection of mosquito mouthparts and digestive tracts from microbial infection during and/or after feeding.

## 1. Introduction

Mosquito salivary glands represent a key organ placed at the interface between mosquito vectors, the environment, mosquito-borne pathogens, and vertebrate hosts. Salivary glands not only produce and secrete saliva, a complex mixture of molecules with biochemical and pharmacological activities [1], but also, as a prerequisite for transmission, are invaded by pathogens (e.g., *Plasmodium* and arboviruses). Saliva plays a crucial role in mosquito feeding and contributes to the efficient acquisition of both sugar and blood meals, which are necessary for energy supply and egg maturation, respectively. Several components of mosquito saliva are known to exert antihemostatic functions by counteracting host coagulation, vasoconstriction, and platelet aggregation [2,3]; these compounds are important during the feeding process and facilitate the intake of a complete blood meal. Mosquito salivary glands and saliva are also involved in the transmission of pathogens of great relevance to human health, such as protozoa of the genus *Plasmodium* and arboviruses, as, for instance, Dengue, Zika, and Chikungunya viruses [4,5]. Typically, pathogens invade and eventually multiply in the salivary glands and are then transmitted via the salivary duct during the next blood meal. Importantly, mosquito saliva is also known to modulate host immune and inflammatory responses. Since pathogens are transmitted to vertebrate hosts, and exposed to their immune systems, in the context of mosquito salivary components and salivary microbiota [6], these immunomodulatory properties of salivary secretions can have profound effects on host infection and disease transmission [4,6,7,8,9]. Recently, the presence of bacteria in mosquito saliva, their transfer to and growth in a mammalian host, and their possible interactions with *Plasmodium* transmission have been documented [10].

Although the role of salivary glands in mosquito immunity has been poorly investigated so far, at least in comparison to key organs and tissues such as the midgut, fat body, and hemocytes, transcriptomic studies indicated that salivary glands produce molecules with immune activities [2,11,12,13], suggesting that saliva represents a first immune defense against microbes. According to experimental evidence, a substantial amount of mosquito saliva is reingested during both sugar and blood feeding [14,15,16], suggesting that antimicrobial peptides (AMPs) and other immune factors may inhibit bacterial growth in the crop, where part of the sugar meal is stored, and in the gut, where the digestion of the blood meal occurs [17,18,19,20]. Members of the lysozyme gene family are expressed in the salivary glands of adult *A. gambiae* mosquitoes of both sexes, indicating a possible role in preventing and controlling bacterial growth [12,21,22]. Furthermore, the presence of AMPs and other immune factors may limit the development of pathogens invading and/or replicating in the salivary glands. The transcriptional activation of anti-*Plasmodium* immune factors in *Anopheles* salivary glands and the induction of antiviral innate immunity signaling pathways in *A. aegypti* salivary glands have been previously reported [23,24,25,26]. Moreover, according to recent works, inter-organ communication among the midgut/salivary glands/hemolymph may take place upon pathogen stimuli; also, the mosquito immune system can discern between local and systemic challenges, activating different transcriptional responses depending on the nature and route of immune stimulation [27,28].

A mosquito’s innate immunity is known to provide protection against a variety of microorganisms (bacteria, viruses, fungi, and protozoa) and humoral innate immunity through the production of AMPs, certainly representing a primary defense mechanism [29,30,31,32]. Indeed, insect AMPs are involved in endotoxin neutralization, the modulation of immune responses, and pathogen killing, and their broad spectrum of action has sparked strong interest in the potential use as therapeutics against antibiotic-resistant microorganisms [33,34,35,36]. Furthermore, the involvement of AMPs in other biological processes such as microbiota homeostasis, lifespan regulation, tumor control, and neurological activities makes them promising candidates for novel biomedical applications [37,38].

Many AMPs are produced as immature prepropeptide precursors of 60–170 amino acid residues that, after a first maturation step involving the removal of the signal peptide (release of propeptides), are then cleaved with proteases to release biologically active (mature) peptides of 6–50 amino acid residues [39,40]. Their encoding genes are often arranged in clusters/sub-clusters, likely originating from gene duplication events, and this structural organization allows for coordinated transcriptional and post-transcriptional regulation and simultaneous expression in response to an immune challenge [41]. The diverse families of insect AMPs can be grouped into three main classes: (i) linear peptides with a hydrophobic α-helical structure (e.g., cecropins); (ii) cysteine-containing peptides with antiparallel β-sheets or loop structures, stabilized via single or multiple disulfide bridges (e.g., defensins); and (iii) proline- and/or glycine-rich peptides [34]. Typically, insect AMPs are rapidly and transiently synthesized by the fat body and circulating hemocytes through the induction of three distinct intracellular signal transduction cascades (Toll, IMD, and JAK-STAT pathways) after the recognition of foreign microorganisms such as Gram-negative and Gram-positive bacteria, yeast, fungi, and viruses [34,41,42,43]. A basal level of immune activation is also maintained by midgut microbiota, whose composition and complexity significantly vary when changes in the environment and feeding habits occur at the larval and adult stages [44]. In response to specific threats of infection, which in mosquitoes occur mainly by the breaking of external barriers or by ingesting a contaminated blood/sugar meal, the immune signaling pathways are activated at both the local (tissues and organs) and systemic (hemocoel) levels, leading to the early production and circulation of protective AMPs [27,28].

Studies on naturally occurring insect antimicrobial peptides date back to at least forty years ago, when Cecropin was identified in the hemolymph of the lepidopteran *Hyalophora cecropia* [45,46]. Since then, the number of identified insect antimicrobial peptides has rapidly increased: currently, 367 of the 3569 AMPs present in the Antimicrobial Peptide Database (APD; https://aps.unmc.edu/home, accessed on 4th January 2024) are of insect origin [39,47]. Although some typical insect AMPs (e.g., defensins and salivary lysozyme) are evolutionarily conserved along the lineage, variability in both the number and type of AMPs are observed at the order, family, and species levels, suggesting an origin from multiple independent evolutionary events [39,41]. For instance, the moricin and gloverin families are only found within the order Lepidoptera [48] while gambicins are restricted to the Culicidae family [1]. This independent evolutionary origin and divergence explain the observed differences in the size, amino acids composition, function, and mechanism of action of insect AMPs. Some AMPs can cause the rapid death of Gram-positive or Gram-negative bacteria, fungi, parasites, encapsulated viruses, and even tumor cells [49]. AMPs are an integral part of a host’s defense barrier against pathogenic invasions in almost all living organisms [50] and their efficacy on a broad spectrum of microorganisms, even antibiotic-resistant strains [40], as well as the lower risk of resistance development [51], makes them very good candidates for pharmacological applications.

According to transcriptomic, proteomic, and genomic studies performed so far, mosquito saliva carries around 100–150 salivary proteins; only a few salivary proteins have had their antihemostatic and immunomodulatory functions identified or hypothesized [1,2,52]. However, a common theme in mosquito salivary repertoires is the presence of a significant fraction (around 30–40%) of putative salivary proteins that have no sequence similarities to any known protein and whose functions remain completely unknown. We hypothesized that some of these orphan salivary genes could encode for AMPs with a role in protecting mosquitoes against microbial infection after sugar or blood meals. In particular, genes specifically expressed or enriched in female salivary glands could encode for AMPs possibly involved in the defense against human skin/blood bacteria, while genes expressed in both male and female salivary glands could be preferentially involved in the response against environmental bacteria. Starting from transcriptome studies on *Anopheles gambiae* [13,22], we previously selected a list of orphan salivary gland-specific or enriched genes and analyzed the transcriptional modulation of 11 candidates following a local (infectious sugar meal) or systemic (intra-thoracic microinjections) bacterial immune challenge in *A. coluzzii* mosquitoes [27]. This analysis provided the first evidence of the involvement of some candidates in mosquito responses against Gram-negative (*Escherichia coli*) and/or Gram-positive (*Staphylococcus aureus*) bacteria. In the present study, aimed at identifying novel salivary components with antimicrobial activities, we analyzed suitable *A. gambiae* orphan salivary proteins/peptides using two different approaches: in vitro transcription/translation and chemical peptide synthesis. While the first approach had limited success, the chemical synthesis allowed for the identification of two peptides inhibiting bacterial growth, with one of them acting in damaging the bacterial surface as indicated with electron microscopy.

## 2. Results

### 2.1. Putative Salivary AMP Selection

To search for putative antimicrobial peptides expressed in mosquito saliva, we screened a catalogue of putative *A. gambiae* salivary proteins that had been previously compiled [13,52]. A list of 11 candidates was obtained, taking into consideration (i) tissue- and sex-specific expression profiles [13,22] and (ii) sequence/structural features as length, hydrophobicity, basic and/or acidic patterns, predicted alpha helices and/or beta-sheets, etc., as previously described in larger detail [27]. The main sequence features of the selected candidates are summarized in Table 1 and a few additional information can be found in Supplemental Tables S1 and S3. To this list of 11 candidates, the salivary lysozyme was added as control [53].

After the removal of the predicted signal peptides, three of the eleven candidates appeared to be shorter than 60 amino acids (hyp13, 34 aa; hyp15, 48 aa; hyp6.2, 58 aa); for these, we decided to proceed to the chemical synthesis. A noteworthy piece of information is that according to prediction analysis performed using the SpiderP tool at the ArachnoServer website (https://arachnoserver.qfab.org/mainMenu.html, accessed on 1 July 2019) [54,55], hyp13 and hyp6.2 may be initially synthesized as propeptides, with mature peptides being 19 and 35 amino acids in length, respectively (Table 1 and Table S3). For these reasons, both the putative propeptides and mature peptides were chemically synthesized. Although the SpiderP tool has been discontinued, propeptide cleavage sites placed at the same positions were also identified using the ProP-1.0 prediction tool (https://services.healthtech.dtu.dk/services/ProP-1.0/ [56], accessed on 1 July 2019), albeit with scores below the threshold. For the remaining eight candidates plus lysozyme, which were over 60 amino acids in length, we opted for expression in recombinant form using a cell-free expression system based on wheat germ lysate and in vitro transcription/translation. We selected this method to benefit from a eukaryotic system (avoiding troubles often encountered when using prokaryotic systems to express putative antibacterial factors) and to produce rapidly small amounts of different recombinant polypeptides suitable for functional screening using bacterial growth inhibition assays. This procedure allowed for the obtaining of variable amounts of recombinant His-tagged peptides/proteins; these were detectable using both Western Blot and, in most cases, Coomassie staining (Figure S1 and Table S2). However, although the proteins were correctly expressed, the yields were low and did not allow for purification by exploiting the His-tag. Bacterial growth inhibition assays were attempted anyway, comparing unpurified in vitro translated products to a control transcription/translation reaction using an empty vector, but the results were only partially reliable and/or reproducible. For this reason, we provide the results of these experiments in the Supplementary Materials (Figure S2) and will discuss in the rest of the manuscript only the results obtained with the chemically synthesized peptides, which were produced in adequate amounts and purity (Table S3).

Putative antimicrobial activities were tested, employing in vitro liquid growth inhibition assays on both Gram-negative (*Escherichia coli* strain ATCC 25922 and *Serratia marcescens* strain ATCC 13880) and Gram-positive (*Staphylococcus aureus* strains ATCC 25923 and ATCC 6538P) bacteria. Peptides were incubated with bacteria in 96-well plates at different concentrations (75–150–300 μM) and, after overnight incubation at 37 °C, aliquots from cultures were (i) used for optical density determination (OD_570_) to evaluate microbial growth and (ii) plated, to count colony-forming units (CFUs) and evaluate bacterial survival. Three independent biological replicates were performed, and statistical analysis was applied to CFU datasets (Figures S3 and S4).

### 2.2. Hyp15: Structural Features and Growth Inhibition Assays

**Figure 1:**
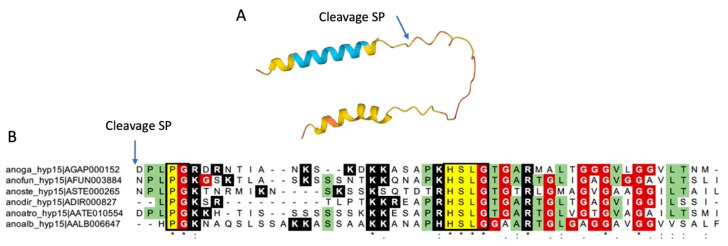
Structural features of the *A. gambiae* hyp15 and sequence conservation among selected anopheline species. (**A**) AlphaFold structure with color-coded confidence metric (pLDDT): Light Blue, confident (90 > pLDDT > 70); Yellow, low (70 > pLDDT > 50); Orange, very low (pLDDT < 50). The predicted signal peptide (SP) cleavage site within the full-length sequence of hyp15 is indicated with the arrow. (**B**) Multiple alignments of hyp15 family members from 6 anopheline species. Fully conserved residues are highlighted in yellow and indicated with asterisks. Conservative (:) and semi-conservative (.) substitutions are also shown. Highlighted in green are the amino acid residues shared among >50% of the aligned sequences. Glycine residues are highlighted in red and positive-charged residues (Arginine and Lysine) in black. Anoga, *Anopheles gambiae*; anofun, *Anopheles funestus*; anoste, *Anopheles stephensi*; anodir, *Anopheles dirus*; anoatro, *Anopheles atroparvus*; anoalb, *Anopheles albimanus*. Sequences were obtained from Additional file 19 of publication [52], which includes FASTA sequences downloaded from VectorBase (https://vectorbase.org/, accessed on 14 June 2023).

Hyp15 is a small protein (full length, 78 aa; without signal peptide, 48 aa) whose expression in *A. gambiae* is highly enriched in adult female salivary glands [13]. It is specifically found in anopheline mosquitoes and does not show significant similarity to any other known protein. The hyp15 protein carries a putative signal peptide according to SignalP-5.0, whereas no propeptide putative cleavage sites were predicted using the SpiderP tool. The mature polypeptide (48 aa, 4.8 kDa) is Cys-free with a pI of 10.55. According to Alpha fold structure prediction, the mature protein, after signal peptide removal, is largely disordered with a low-confidence alpha helical structure corresponding to the C-terminal Gly-rich region (Figure 1A). The alignment of hyp15 from a few anopheline family members (Figure 1B) showed a highly conserved PLPG tetrapeptide at the N-terminus, followed by a positively charged region, a fully conserved HSLG tetrapeptide, and a glycine-rich carboxy terminus [52]. Preliminary analyses showed no significant inhibition of bacterial growth after the incubation of the synthetic hyp15 peptide at a concentration of 150 µM with both Gram-negative *E. coli* and Gram-positive *S. aureus* (Table S3 and Figure S3) bacteria. For these reasons, the hyp15 polypeptide was not analyzed further.

### 2.3. Hyp6.2: Structural Features and Growth Inhibition Assays

**Figure 2:**
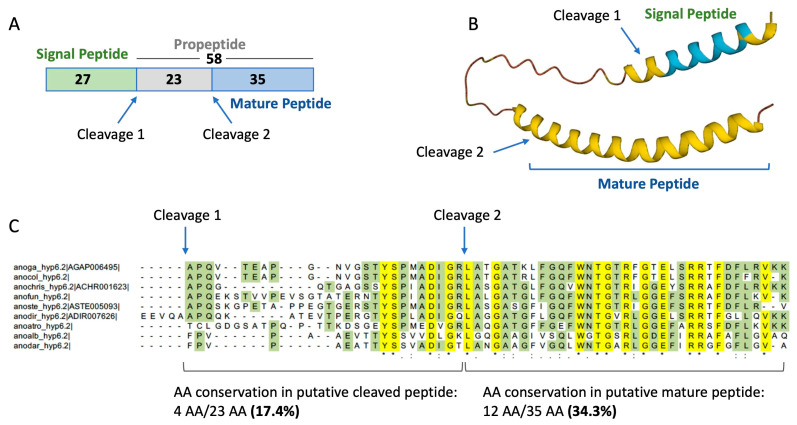
Structural features of the *A. gambiae* hyp6.2 and sequence conservation among selected anopheline species. (**A**) Predicted cleavage sites within the full-length sequence of hyp6.2 are indicated with arrows (numbers indicate amino acidic sequence lengths). (**B**) AlphaFold structure with color-coded confidence metric (pLDDT): Light Blue, confident (90 > pLDDT > 70); Yellow, low (70 > pLDDT > 50). (**C**) Multiple alignments of hyp6.2 family members from 9 anopheline species. Fully conserved residues are highlighted in yellow and indicated with asterisks. Conservative (:) and semi-conservative (.) substitutions are also shown. Highlighted in green are the amino acid residues shared among >50% of the aligned sequences. Anoga, *Anopheles gambiae*; anocol, *Anopheles coluzzii*; anochris, *Anopheles christyi*; anofun, *Anopheles funestus*; anoste, *Anopheles stephensi*; anodir, *Anopheles dirus*; anoatro, *Anopheles atroparvus*; anoalb, *Anopheles albimanus*; anodar, *Anopheles darlingi*. Sequences were obtained from Additional file 19 of publication [52], which includes FASTA sequences downloaded from VectorBase (https://vectorbase.org/, accessed on 14 June 2023).

Hyp6.2 (AGAP006495) is an intronless gene located on chromosome 2L in a genomic region, including also hyp8.2 (AGAP006494, Table 1) and other salivary genes (SG2, SG2b, and SG3) [52]. The hyp6.2 propeptide (full length, without signal peptide) has a molecular weight of ~6.3 kDa (58 aa), does not carry any cysteine, and is basic (pI 10.41). The putative mature form predicted using the SpiderP tool (molecular weight ~4.0 kDa, 35 aa, Figure 2A) has an even more basic predicted pI (11.57, Arg + Lys 20%). Both forms are relatively enriched in glycine (11.4–12.1%). Structural prediction indicates that the mature peptide may adopt an alpha helical structure for almost its entire length (Figure 2B). According to BLAST searches against non-redundant databases and genomic analyses [52], hyp6.2 is an anopheline-specific protein with no similarity to any known protein from other species. Notably, the alignment of hyp6.2 proteins from representative anopheline species show a remarkable conservation of the putative mature forms (12 invariant positions over 35 amino acid residues, 34.3%), significantly higher as compared to the putative precursor (4 invariant positions over 23 amino acid residues, 17.4%; Figure 2C).

**Figure 3:**
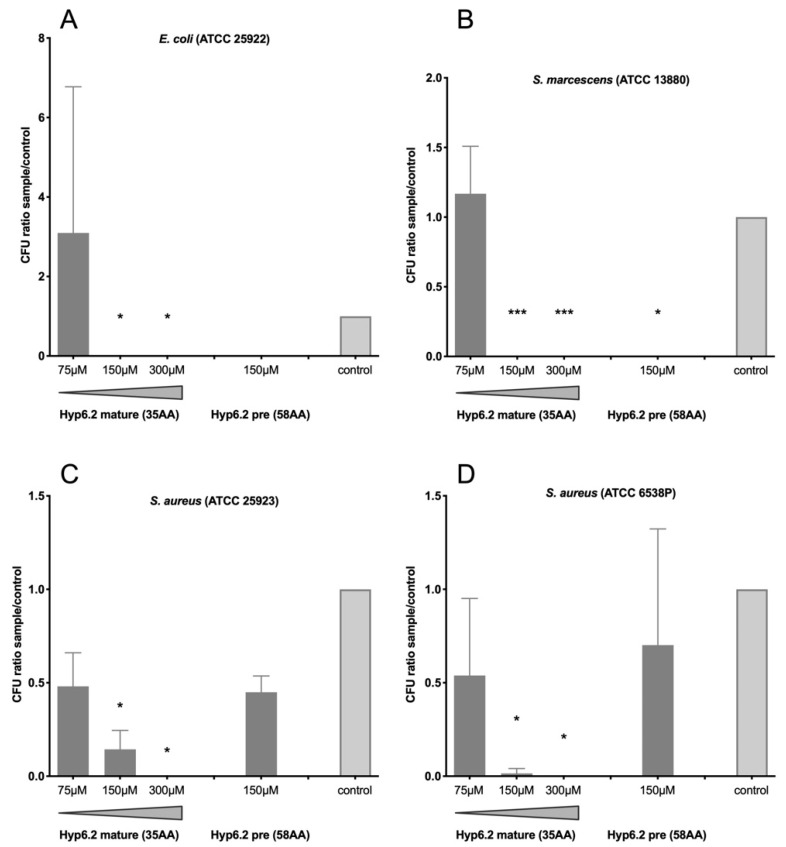
Bacterial growth inhibition with the mature and propeptide precursor (pre) forms of hyp6.2. The bacterial growth of Gram-negative *Escherichia coli* ATCC 25922 (**A**) and *Serratia marcescens* ATCC 13880 (**B**) and of Gram-positive *Staphylococcus aureus* ATCC 25923 (**C**) and ATCC 6538P (**D**) was evaluated by counting CFU after incubation overnight at 37 °C in the presence or the absence (control) of the two forms of the hyp6.2 peptide. Peptide concentrations are reported below the x-axis. CFU ratios between samples and controls are reported, and they represent the means with standard deviations of three independent experiments. CFU values were analyzed using 2-way ANOVA followed by Dunnett’s multiple comparisons test to assess the statistical significance of samples in comparison to the controls. *: *p* < 0.05; ***: *p* < 0.001.

The growth of both Gram-positive and Gram-negative bacteria was significantly inhibited when incubated with the mature shorter form of hyp6.2 at different concentrations. Indeed, the incubation of the mature hyp6.2 peptide with Gram-negative bacteria determined a strong to total inhibition of microbial growth using concentrations of 300 μM and 150 μM (Figure 3A,B), while treatments at 75 µM did not show any significant effect on bacterial growth and survival as compared to control. The inhibition of both *E. coli* and *S. marcescens* was also detectable using the long, immature form of hyp6.2 at 150 μM, even though the statistical significance was restricted only to *S. marcescens* (Figure 3B). A slightly lower yet significant inhibition was observed incubating the mature form of hyp6.2 with both Gram-positive *S. aureus* strains (Figure 3C,D) at 300 and 150 μM; an approximately 50% reduction in the numbers of colonies could also be detected in incubating bacteria with the mature form of hyp6.2 at 75 μM or with the longer immature form at 150 µM but these changes did not reach statistical significance when compared to controls. Similar growth inhibition rates were observed in measuring liquid culture optical density at 570 nm (Figure S4). Overall, both forms of hyp6.2 strongly inhibited the growth of the Gram-negative bacterial strains tested at a concentration of 150 µM, with an apparently stronger effect on *S. marcescens* than *E. coli*. The inhibitory effects on the two Gram-positive *S. aureus* strains were somehow less pronounced and reached statistical significance only when the mature shorter form of hyp6.2 was used, suggesting that the two forms may exert different antimicrobial activity, at least on *S. aureus*.

### 2.4. Hyp13: Structural Features, Growth Inhibition Assays, and Electron Microscopy

**Figure 4:**
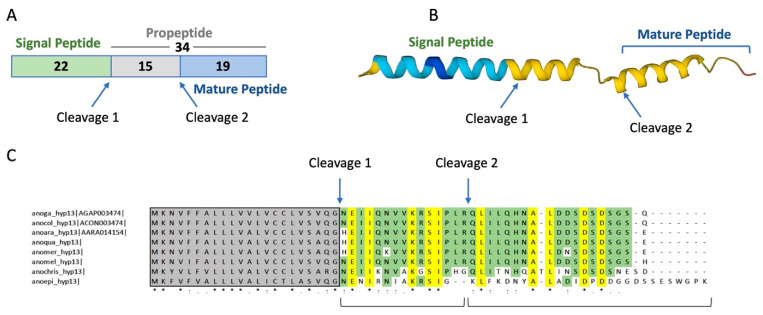
Structural features of the *A. gambiae* hyp13 and sequence conservation among selected anopheline species. (**A**) Predicted cleavage sites in the full-length sequence of hyp13 are indicated with arrows (numbers indicate length in amino acids). (**B**) AlphaFold structure with color-coded confidence metric (pLDDT): Blue, very high (pLDDT > 90); Light Blue, confident (90 > pLDDT > 70); Yellow, low (70 > pLDDT > 50). (**C**) Multiple alignments of hyp13 orthologues in anopheline species belonging to the Pyretophora series (i.e., the *A. gambiae* species complex, *A. christyi*, and *A. epiroticus*). Fully conserved residues are highlighted in yellow and indicated with asterisks. Conservative (:) and semi-conservative (.) substitutions are also shown. Highlighted in green are the amino acid residues shared among >50% of the aligned sequences. Anoga, *Anopheles gambiae*; anocol, *Anopheles coluzzii*; anoara, *Anopheles arabiensis*; anoqua, *Anopheles quadriannulatus*; anomer, *Anopheles merus*; anomel, *Anopheles melas*; anochris, *Anopheles christyi*; anoepi, *Anopheles epiroticus*. Sequences were obtained from Additional file 19 of publication [52], which includes FASTA sequences downloaded from VectorBase (https://vectorbase.org/, accessed on 14th June 2023).

The gene encoding hyp13 (AGAP003474) is located on chromosome arm 2R:15C and shows an almost ubiquitous expression pattern with higher levels in both male and female adult salivary glands [13,52]. Hyp13 encodes for a 34-amino acid peptide of ~3.8 kDa (34 aa), with a predicted pI of 4.75. The putative mature form (Figure 4A) has a molecular weight of ~2.0 kDa (19 aa) and an even more acidic predicted pI of 3.77. Peptide structure prediction software suggested the presence of two alpha helices (Figure 4B) with the putative mature peptide being composed of an alpha helix followed by a short random coiled region. Orthologues of hyp13 could be identified only in *Anopheles* mosquitoes of the Pyretophora series (i.e., the species belonging to the *A. gambiae* complex with the addition of *A. christyi* and *A. epiroticus*, Figure 4C) where they share a 41–59% (hyp13) amino acid residue identity [52].

**Figure 5:**
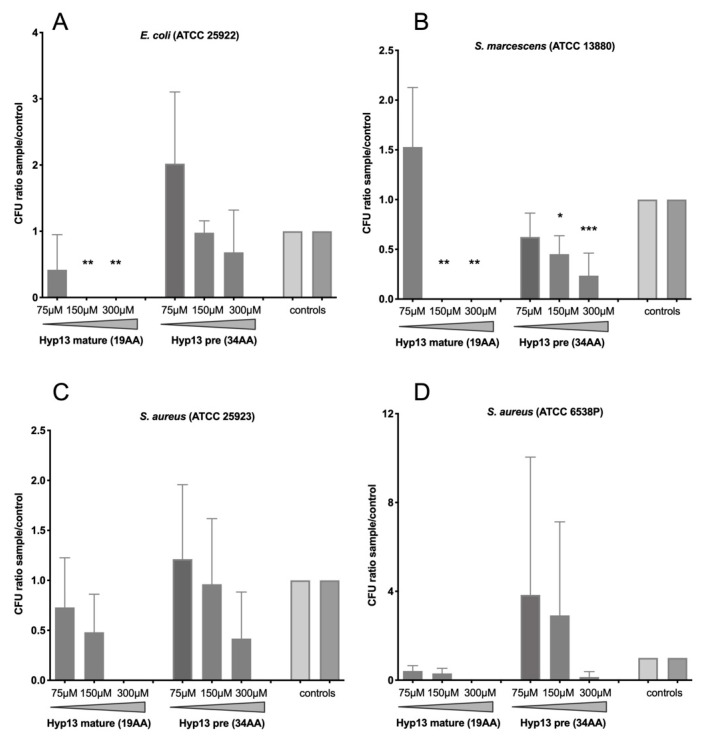
Growth inhibition via the mature and propeptide precursor (pre) forms of hyp13. Bacterial growth of the Gram-negative *Escherichia coli* ATCC 25922 (**A**) and *Serratia marcescens* ATCC 13880 (**B**) and of the Gram-positive *Staphylococcus aureus* ATCC 25923 (**C**) and ATCC 6538P (**D**) was evaluated by counting CFU after incubation overnight at 37 °C in the presence or the absence (control) of the two forms of the hyp13 peptide. Peptide concentrations are reported below the x-axis. CFU ratios between samples and controls are reported, and they represent the means with standard deviations of three independent experiments. CFU values were analyzed using 2-way ANOVA followed by Dunnett’s multiple comparisons test to assess the statistical significance of samples in comparison to the controls. *: *p* < 0.05; **, *p* < 0.01; ***: *p* < 0.001.

The growth of Gram-negative bacteria was significantly inhibited when incubated with the mature, short form of hyp13 at concentrations of 300 μM and 150 μM (Figure 5A,B), while a lower concentration (75 μM) did not show significant effects, even though a colony decrease of around 50% could be detected in *E. coli*. The significant growth inhibition of *S. marcescens* (but not of *E. coli*) was also observed using the long, immature form of hyp13 at 300 μM (70–80% inhibition) and 150 μM (50%), but the effects were clearly less evident than with the short form (Figure 5B). No significant inhibition of bacterial growth caused by the precursor or the mature form of hyp13 was observed on the Gram-positive *S. aureus* strains, although some dose-dependent reduction in CFU numbers could be observed (Figure 5C,D). Similar growth inhibition rates were observed by measuring liquid culture optical densities at 570 nm (Figure S4). In summary, the putative mature form appeared more active than the putative propeptide form of hyp13, both on the Gram-negative and on the Gram-positive bacterial strains tested, with inhibitory growth effects reaching statistical significance only on Gram-negative *E. coli* and *S. marcescens* bacteria.

**Figure 6:**
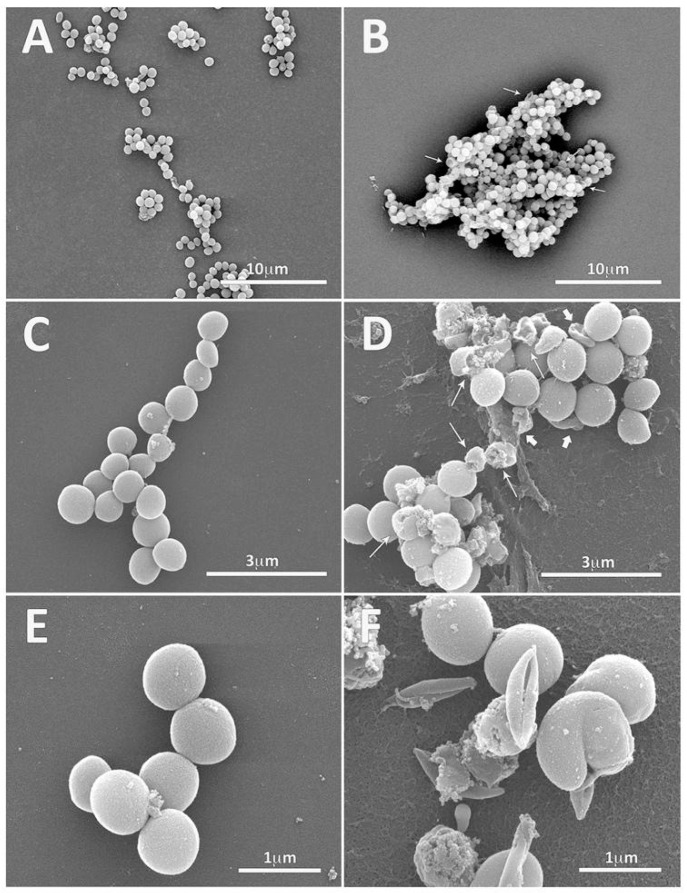
Electron microscopy images of *S. aureus* bacteria treated with hyp13. Panels (**A**,**C**,**E**): control, untreated bacteria. Panels (**B**,**D**,**F**): *S. aureus* bacteria treated with 100 μM of hyp13’s short, mature form. Scale bars are reported in each panel. Thick arrows highlight membrane defects, while thin arrows point to the presence of holes in the bacterial surface and the leakage of the cellular content.

At the morphological level, scanning electron microscopy was used to analyze the effects of the short mature form of hyp13 on the Gram-positive *S. aureus* ATCC 25923 and the Gram-negative *E. coli* ATCC 25922 strains. The scanning electron microscopy (SEM) results of untreated cells showed a regular bacterial surface with no discernible ultrastructural changes of both *S. aureus* and *E. coli*. Untreated *S. aureus* bacterial cells were healthy with a nearly spherical shape and smooth intact surfaces (Figure 6A,C,E); a certain number of cells appeared as aggregates, typical of the staphylococcus genus that can grow in clusters, and septa were present in the actively dividing stage. A sub-MIC concentration of 100 μM of hyp13 was chosen for SEM because this concentration is unable to kill or destroy all bacterial cells according to CFU counts from growth inhibition experiments; indeed, 50% and 65% reductions in the survival of *S. aureus* ATCC 25923 and ATCC 6538P, respectively, were observed in growth inhibition assays using the mature form of hyp13 at 150 μM. Scanning electron micrographs of hyp13-treated *S. aureus* bacteria showed several cells with extensive surface damage and distorted cell morphology (Figure 6B,D,F). Membrane distortions with surface depressions and biconcave appearances were visualized (Figure 6D, thick arrows). The formation of holes on the cell surface and the leakage of cellular content were also visible (Figure 6B,D, thin arrows). The micrographs at higher magnification better revealed the cell surface roughness, deep perforations, or holes, along with complete lysis, and the collapse and shrinkage of several cells and cell debris (Figure 6D,F). From extensive SEM observations, it was possible to reveal that peptide treatment also induced many bacterial cells to aggregate in clusters with a higher number and size compared to the untreated cells. This could contribute to the inhibitory effect of the hyp13 peptide found through CFU counts, in addition to the direct damage of the bacterial surface.

**Figure 7:**
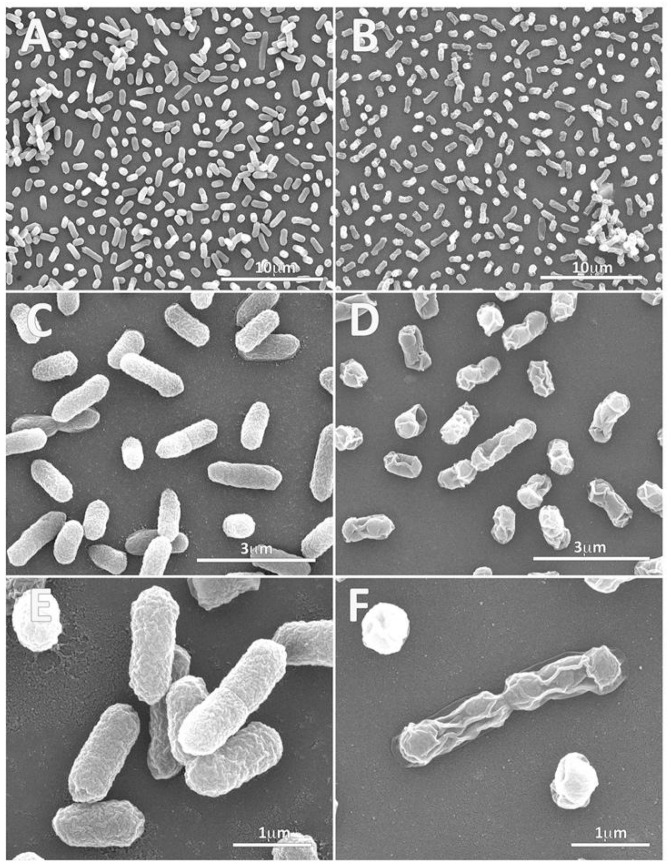
Electron microscopy images of *E. coli* bacteria treated with hyp13. Panels (**A**,**C**,**E**): control, untreated bacteria. Panels (**B**,**D**,**F**): *E. coli* bacteria treated with 100 μM of hyp13’s short, mature form. Scale bars are reported in each panel.

Also, the SEM analysis of untreated *E. coli* showed cell surfaces that were normal, uniform, complete, rough, and full (Figure 7A,C,E). In contrast, treated *E. coli* cells showed significant ultrastructural alterations, including distorted and deformed characteristics (Figure 7B,D,F) that were not seen in the control. Almost all the treated cells showed deep changes in the structure, quite similar to those found in “bacterial ghosts”. Deformed bacteria retained the same size and the overall morphology as the intact and living bacteria. The hyp13 peptide treatment yielded a change in the surface texture with a smoothing of the bacterial surface. Many cells showed the cell membrane shrank into the lumen of the cell, while other cells displayed cell membrane collapsed inward as if the cell contents were all expelled. Collapsed membranes were not randomly distributed over the envelope but were mainly found at the equatorial center, poles, or positions near the poles of the bacteria. Since growth inhibition assays revealed only a partial inhibitory activity of hyp13 at 75 μM, while 100% bacterial survival inhibition was detected at 150 μM, it is possible to hypothesize that shrunken bacteria with no deep depressions on the cell surface were still alive at the sub-MIC 100 μM concentration used. Conversely, bacteria showing more dramatic changes in surface morphology, with a flattened and shrunken appearance along with a collapsed structure perhaps representing dead or dying bacterial cells.

## 3. Discussion and Conclusions

Mosquito saliva is well known for its ability to counteract host hemostasis and modulate the immune and inflammatory responses of vertebrates to tissue injury [1,2,18]. In this regard, the involvement and the role of some salivary proteins has been clarified [2] and the possible contribution of small non-coding RNAs has been suggested [57,58,59], corroborating the existence of a complex network of molecules mediating the communication and the interplay between the vector and human host cells. Moreover, salivary glands are specifically invaded by pathogens before transmission to the human host [60,61,62] and salivary-secreted molecules also modulate pathogen transmission and host infection [8,9]. However, on the vector side, the possible role of salivary factors in the defense against salivary gland invasion by pathogens has been poorly investigated so far, with only few proofs that a mosquito’s innate immunity might play a local and specific role at this stage of pathogen’s lifecycle [4,29,42].

Investigating the complexity of salivary components posed the question of whether saliva might contain antimicrobial activities protecting the mouthparts and the initial phases of blood- or sugar-feeding processes. In fact, salivary proteins are reingested by blood-feeding insects [14,15,63] and, therefore, could be involved in minimizing the bacterial growth in both sugar meal (stored in the crop) and blood meal (digested in the gut). In this work, we started a search for antimicrobial activities in mosquito saliva and, employing a catalogue of *A. gambiae* salivary proteins, we selected a short list of candidates according to the following criteria: (i) the absence of sequence similarity to any known protein; (ii) the presence of sequence/structural features typical of antimicrobial peptides; and (iii) the tissue- and sex-specific expression profile. This selection resulted in a list of 11 candidates and, according to their length, two different strategies were used in order to proceed to a functional screening. Polypeptides over 60 amino acids in length were obtained using a eukaryotic cell-free transcription-and-translation system to avoid the limits and obstacles of prokaryotic expression; however, the low yields obtained with this approach hampered any attempt of recombinant protein purification from cell-free extracts. Despite this limitation, pilot experiments were performed with crude extracts including the recombinant polypeptides and provided some preliminary indications about a possible antimicrobial activity of hyp12 against *E. coli*, a finding that may deserve future investigation. No bacterial growth inhibition was observed with the other recombinant polypeptides (Supplementary Materials); however, their low concentration (estimated range of 3–13 μM) and/or interfering components from the crude extracts may have contributed to this result. On the other side, candidates shorter than 60 amino acid residues (hyp6.2, 58 aa; hyp13, 34 aa; hyp15, 48 aa) were obtained in amounts suitable for functional assays via chemical synthesis. Since the presence of putative propeptide cleavage sites was predicted for hyp6.2 (putative mature form, 35 aa) and hyp13 (putative mature form, 19 aa), for these two peptides, both forms were synthesized. The comparative sequence analysis of hyp6.2 among different *Anopheles* species showed a significantly higher degree of sequence conservation in the amino-terminal portion, which corresponds to the predicted mature peptide (Figure 2). This may be the result of a selective pressure acting on the putative functional mature portion of the peptide; moreover, in a large proteomic analysis [64], a single peptide from hyp6.2 was identified in the salivary glands of *A. gambiae*, and this peptide (LFGQFWNTGTR) maps to the putative mature form of hyp6.2. These observations provide indirect support for the predicted propeptide cleavage site in hyp6.2. It is worth emphasizing that transcriptional induction through a bacterial or parasite challenge has been previously reported for two of these three peptides, namely hyp6.2 and hyp13. Indeed, the hyp6.2 transcript appeared increased a few hours after an *S. aureus* challenge via infectious sugar meal, suggesting its possible involvement in early local immune responses to Gram-positive bacteria [27]. Hyp13 was not regulated through bacterial systemic or local challenges [27] but was transcriptionally activated with *Plasmodium berghei* 24–28 h post-infectious blood meal [65].

A functional screening was performed using growth inhibition assays, and two Gram-negative (*E. coli* ATCC 25922 and *S. marcescens* ATCC 13880) and two Gram-positive (*S. aureus* ATCC 29213 and 6538P) bacterial strains were selected. The choice of these bacteria was mainly due to their widespread employment in studies investigating mosquito innate immunity and the activation of immune pathways, including AMP transcriptional activation. These microbic models may also partially mimic the environment that the mosquito may find during feeding. Indeed, members of the *Staphylococcus* genus are among the most common bacteria in the human skin microbiome [66], with *S. aureus* known to colonize this location [67]. On the other side, although *E. coli* and *Serratia* are rarely found on human skin, *E. coli* is a common model for studies on plant-bacteria communities [68].

The growth of both Gram-positive and Gram-negative bacteria was significantly inhibited by the mature, shorter form (35 aa) of hyp6.2 at 300 and 150 µM, whereas the putative propeptide (58 aa) showed an effect only on the growth of Gram-negative bacteria at 150 µM. A noteworthy piece of information, as previously mentioned, is that hyp6.2 transcripts were induced in vivo following an *S. aureus* challenge via an infectious sugar meal [27]. On the other side, neither the short (19 aa) nor the long (34 aa) form of hyp13 significantly reduced the growth of Gram-positive bacteria, even though a dose-dependent decrease could be observed with the shorter mature peptide. Conversely, the significant growth inhibition of the Gram-negative *E. coli* and *S. marcescens* was observed after incubation with the mature form of hyp13 at 300 and 150 µM, and a 50% reduction in *E. coli* growth was also observed at 75 µM. A dose-dependent growth inhibition effect was also found on *S. marcescens* with the putative hyp13 precursor (34 aa). In summary, a dose-dependent effect of the mature forms of hyp6.2 (against both Gram-negative and Gram-positive bacteria) and hyp13 (against Gram-negative bacteria) was observed. Hyp6.2 propeptide was also active to a lesser extent, while almost no activity could be observed when the long form of hyp13 was tested. In our experimental conditions, the putative MIC of these peptides can be placed between 75 and 150 µM, which is around ten times higher as compared to the antimicrobial activity of other mosquito AMPs (such as, for instance, cecropins and defensins). This observation raises questions concerning the possible role of these two peptides in physiological conditions, an issue that deserves some speculation.

In this respect, evaluating the amounts of small peptides such as hyp6.2 (4.05 kDa) and hyp13 (2.04 kDa) in the saliva of *Anopheles* mosquitoes is not straightforward. A tentative indirect estimation may be performed considering transcript abundance, as determined using RNA-seq, and using as a comparison the salivary protein gSG6 (AGAP000150) [69]. This protein may be an appropriate reference for its low molecular weight (10.07 kDa) and because it was clearly visible when protein extracts from 20 pairs of female *A. gambiae* salivary glands were stained using Coomassie after fractionation with SDS-PAGE [70]. Assuming that the stained band corresponded to 0.5 µg of gSG6 protein, we can estimate that saliva from a single mosquito may carry around 25 ng of gSG6. During feeding, a mosquito discharges approximately half of the salivary gland protein content [71,72] and secretes around 1.5 nanoliters of saliva [73,74]. In this scenario, an *Anopheles* mosquito may secrete, while feeding, 12.5 ng of gSG6 at a concentration of 8.3 ng/nL, which would roughly correspond to 775 µM. According to an RNA-seq study on *A. gambiae* salivary glands [75], gSG6 transcripts are 6.7 and 27 times more abundant as compared to hyp13 and hyp6.2, respectively [Transcripts per Million (TPM): gSG6, 2509; hyp13, 373; hyp6.2, 93]; notably, very similar ratios (4.8 and 26) were found retrieving data from a different RNA-seq study on *A. gambiae* salivary glands infected with *Plasmodium falciparum* [76]. Assuming that similar ratios are conserved at the protein level, hyp13 may reach a concentration of 130 µM in the saliva of *A. gambiae* females, and hyp6.2 values of around 30 µM. These concentrations may be fully compatible with a role in limiting bacterial growth at the level of secretory cavities, salivary ducts, and mouthparts. On the other side, taking into account that the average size of a sugar meal is around 0.8 µL and of a blood meal 2–3 µL [77,78,79], the concentrations that hyp6.2 and hyp13 may reach in physiological conditions still appear far from the estimated MIC. However, we should consider that technical reasons and/or specific experimental conditions may have led to an underestimation of the antimicrobial capacity of these peptides in our growth inhibition assays. Moreover, in natural conditions, these peptides may act in concert with other components secreted in the crop or in the midgut and, therefore, may be effective in limiting bacterial growth in the sugar or blood meal at lower concentrations.

Additional information was obtained via electron microscopy studies on the short form of hyp13. This peptide was chosen because, in comparison to the other peptides, it showed a higher dose-dependence effect, a clearer specificity of action (Gram-negative bacteria are more affected than Gram-positive), and a more pronounced difference between putative precursor and mature forms. According to the ultrastructural features observed using SEM, the short form of hyp13 induced, in some way, membrane disruption in both Gram-positive and Gram-negative bacteria. In *E. coli*, the effect was more evident, with the peptide treatment triggering dramatic ultrastructural changes and irreversible alterations leading to bacterial ghosts [80,81] and death in most cells. In accordance with these observations, as reported for other peptides [82,83], hyp13 may exert its antibacterial action in *E. coli* acting as a cell-penetrating peptide and causing lysis throughout pore formation after binding to the outer membrane. Peptide insertion would lead to a transmembrane channel causing membrane perturbation, visualized as the deep depression in membranes at the poles and center of bacteria with the loss of cytoplasmic material. Based on the comparison of the SEM images and CFU counts, it appears that this activity is exerted after the accumulation of membrane defects that destroy the surface and ultimately break down the integrity of bacteria. After 24 h of peptide treatment at sub-MIC concentrations (100 microM), most of the *E. coli* cells appeared deeply deformed, as visualized with SEM, but still alive as revealed with the bacterial killing assay. It cannot be ruled out that after hyp13 binds with outer membranes, a critical peptide concentration and/or aggregation is required to be achieved on the membrane to lead to the outer membrane disruption and subsequent interaction with the inner membrane leading to bacterial lysis. The effects of hyp13 on *S. aureus* morphology appeared milder as compared to *E. coli* and suggest that more than one mechanism might be involved in hyp13’s antibacterial activity, perhaps because of the different cell wall compositions of Gram-positive bacteria. In fact, only a relatively small number of bacterial cells appeared destroyed and deeply damaged, which agrees with CFU counts showing that a higher peptide concentration was required to achieve a significant reduction in bacterial viability. It is not unlikely, as observed for other peptides [84], that hyp13 may be entrapped by some components of the cell wall, as teichoic acids, that may act as “scavengers” for the peptide, decreasing the local peptide concentration on the cytoplasmic membrane and preventing full membrane coverage and, in turn, membrane disruption. In this scenario, bacterial killing via membrane disruption would occur only when the membrane surface is fully saturated with hyp13 peptide. Furthermore, the higher aggregation rate found in the hyp13-treated samples suggests that the peptide bound to the bacterial surface may mediate the interaction between bacterial cells, explaining the large and irregular bacterial clusters observed with SEM. Notably, this mechanism may contribute to the antibacterial effect since bacterial aggregation prevents bacteria from binding to several different cell tissues and promotes their removal [85]. The understanding of the exact modes of action of novel AMPs represents a crucial passage in their evaluation as potential pharmacological agents. AMPs’ mechanism of action can be distinguished between (i) membrane-targeting AMPs, which impair the structural integrity of the cell membrane and (ii) non-membrane-targeting AMPs, mainly acting by targeting the synthesis of nucleic acids, essential enzymes, or other functional proteins [49]. Also, it was suggested that bacterial death caused by AMPs could be a result of multiple actions, known as a multi-hit mechanism, thus increasing the efficiency of AMPs and evading resistance development. The mode of action of each AMPs likely varies depending on several parameters such as the peptide concentration, tissue localization, and growth phase of the specific bacterial species faced. Importantly, regardless of the exact mechanism of action and the specific target site, the antibacterial activity of AMPs is dependent on the interaction with microbial membranes [40,51].

In conclusion, searching for antimicrobial activities in the salivary glands of the malaria mosquito *A. coluzzii* brought the identification of two short salivary peptides, hyp6.2 and hyp13, which can limit the growth of Gram-positive and Gram-negative bacteria with an MIC of around 100 µM and may contribute to the immune protection of mosquito mouthparts and digestive tracts during blood and sugar feeding.

## 4. Materials and Methods

### 4.1. Sequence Retrieval and Bioinformatic Tools

The sequences of genes listed in Table 1 and Table 2 were retrieved from VectorBase (https://vectorbase.org/, accessed on 1 July 2019). The following online software was interrogated to achieve sequence predictive analyses: Expasy (https://www.expasy.org, accessed on 1 July 2019); ProP-1.0 tool [56] (https://services.healthtech.dtu.dk/services/ProP-1.0/, accessed on 1st July 2019); SpiderP tool [54] at ArachnoServer (now dismissed: http://www.arachnoserver.org/spiderP.html, accessed on 1 July 2019); and AlphaFold [86] at EBI (https://alphafold.ebi.ac.uk, accessed on 14 June 2023).

### 4.2. Cloning Procedures for In Vitro Peptide Synthesis

Candidate sequences were cloned into the RTS pIVEX Wheat Germ plasmid (5 Prime GmbH, Hilden, Germany) to promote the transcription and translation of the peptides in a cell-free system. PCR amplicons were generated via RT-PCR from *A. coluzzii* total RNA, digested with SmaI (25 °C) and NcoI (37 °C), and ligated (3:1 ratio) to the linearized pIVEX 1.3 WG vector (5 Prime GmbH, Hilden, Germany). Primer pairs used for RT-PCR amplifications are reported in Table 2. The ligation mixture was used to transform competent *E. coli* DH5α cells and then plated on Luria Broth-agar plates with ampicillin [100 μg/mL] and incubated at 37 °C overnight. Single colonies corresponding to recombinant clones were inoculated in liquid cultures and plasmid DNA was isolated using the PureLinkTM HiPure Plasmid DNA purification kits (Invitrogen ThermoFisher, Waltham, MA, USA). Recombinant clones were verified both via restriction analysis and DNA sequencing.

### 4.3. Peptide Expression in the Wheat Germ Cell-Free System

The synthesis of candidate recombinant peptides was performed through the RTS Wheat Germ CECF System (5 Prime GmbH, Hilden, Germany), a eukaryotic cell-free transcription/translation system based on wheat germ lysate. The “Feeding Solution” and “Reaction Solution” were reconstituted and prepared according to the manufacturer’s protocol. For the small-scale synthesis (WG-100 µL batch format), 15 μL of plasmid DNA (100–300 ng/μL) was mixed with 35 µL of Reaction Solution (15 μL of Reaction Mix, 4 μL of amino acids, 1 μL of Met, and 15 μL of Wheat Germ lysate) to reach a final volume of 50 μL. Afterwards, the Reaction Solution containing plasmid DNA (50 μL) and the Feeding Solution (1 mL, containing 900 μL of Feeding Mix, 80 μL of amino acids, and 20 μL of Met) were aliquoted in their respective chambers (reaction and feeding chamber). One module for each gene was placed in a thermomixer (Eppendorf Thermomixer Comfort, Eppendorf, Hamburg, Germany) and the reactions were incubated at 24 °C, 900 rpm for 24 h. The synthesis of a subset of candidate genes was also performed using the large-scale wheat germ kit (WG-500 µL batch format) and in this case, the plasmid concentration and the feeding/reaction solution amounts were adapted according to the manufacturer’s instructions.

### 4.4. Coomassie Staining and Western Blot Analysis

Sodium Dodecyl Sulphate-PolyAcrylamide Gel Electrophoresis (SDS-PAGE) followed by Coomassie staining or Western blot analysis was performed to confirm the synthesis of the peptides. For each candidate peptide, 1 μL of WG expression reaction solution (from a total volume of 50 μL) was mixed with 4 μL of 5× Loading Buffer and 15 μL of ddH_2_O. After boiling (98 °C for 10 min), aliquots of 10 μL were loaded on two different gradient gels (4% Stacking/15% Resolving) used for Coomassie staining and Western blot detection, respectively. For Coomassie detection, the gel was fixed for 30 min in 40% MeOH plus 10% acid acetic, rinsed for 5 min with ddH_2_O (three times), stained for 1 h with Coomassie (BioSafe G-250 Bio-Rad, Hercules, CA, USA), and destained with ddH_2_O. Regarding Western blot detection, proteins were transferred to nitrocellulose membrane through electroblotting (1 h at 288 V). The NC membrane was blocked for 1 h (5% low-fat dry milk/0.1% Tween/1XPBS), stained for 30 min with 1:5000 HRP Mouse Anti-6xHis monoclonal antibody, and washed with PBST (PBS-Tween 0.1%) 3 times. Finally, the membrane was incubated for 1 min with Chemio-luminescent Peroxidase Substrate (Sigma-Aldrich, St. Louis, MO, USA) and then exposed, typically for 2–5 min.

### 4.5. Chemical Synthesis of Peptides

The peptides hyp15; hyp13—long form (putative propeptide); hyp13—short form (putative mature); hyp6.2—long form (putative propeptide); and hyp6.2—short form (putative mature) were chemically synthesized using GenScript (GenScript Biotech, Piscataway, NJ, USA). The peptides were resuspended in the appropriate solvent according to their biochemical properties: endotoxin-free ultrapure water (hyp15 and hyp13 long forms), DMSO (hyp6.2 short and long forms), and 3% ammonia (hyp13 short form). The sequence, molecular weight, length, purity, and solubility properties are reported in Supplemental Table S3.

### 4.6. Bacterial Strains

Bacterial strains were obtained from the American Type Culture Collection (ATCC; Rockville, MD, USA) and include the Gram-negative strains *Escherichia coli* (ATCC 25922) and *Serratia marcescens* (ATCC 13880), and two Gram-positive strains of *Staphylococcus aureus* (ATCC 25923 and ATCC 6538P). The microorganisms were grown in Luria Broth (Oxoid, ThermoFisher, USA) at 37 °C and stored in 15% glycerol-BHI at −80 °C.

### 4.7. Bacterial Growth Inhibition Assay

To screen for recombinant peptides with potential antimicrobial activity, single colonies of a Gram-negative (*E. coli* ATCC 25922) or a Gram-positive (*S. aureus* ATCC 25923) strain were inoculated in 15 mL of Luria Broth and incubated overnight in a shaking incubator at 225 rpm at 37 °C. Bacterial suspensions were diluted to 0.4 OD_600_ and used for growth inhibition assays, which were performed in 96-well plates (non-treated, Starstedt Numbrecht, Nümbrecht, Germany), while the bacterial growth was monitored in the presence or absence of the peptides in a microplate reader (BioTek Synergy HT, BioTek Instruments, Winooski, VT, USA). Ampicillin and Cecropin (synthetic CecA, Sigma-Aldrich) were used as positive controls, while crude extract obtained from protein synthesis with the empty pIVEX vector were employed as negative controls. Briefly, each well of the plate was loaded with 5 μL of bacterial culture, 75 μL of Luria Broth, and 20 µL of (i) crude WG extract with peptide expressed from recombinant pIVEX vector, (ii) crude WG extract with the empty pIVEX vector (negative control), (iii) ddH_2_O (negative control), or (iv) Amp or Cec 1.25 μg/μL (positive controls), to obtain a final volume/well of 100 μL. Bacterial growth was monitored in a BioTek Synergy HT microplate reader according to the following protocol: incubation overnight at 37 °C with a shaking for 2 min every 10 min and an OD measurement at 570 nm every 10 min for 24 h. Similar bacterial growth inhibition assays were performed to test the potential antimicrobial activity of chemically synthesized peptides (hyp6.2 short and long forms, hyp13 short and long forms, and hyp15).

### 4.8. Minimum Inhibitory Concentration (MIC) and Minimum Bactericidal Concentration (MBC)

The sensitivity of *E. coli* (ATCC 25922), *S. marcescens* (ATCC 13880), and *S. aureus* (ATCC 25923 and ATCC 6538P) to the selected peptides (mature and pre-mature forms of both hyp6.2 and hyp13 peptides) was determined in 96-well plates by inoculating the bacteria in Luria Broth containing serial dilutions of the peptides. After 24 h of incubation, the MIC value was determined both by measuring the OD at 600 nm and by plating on Luria Broth agar plates. To determine the MBC values, defined as the lowest concentration that kills 99.9% of the bacteria in the initial inoculum, 10 μL of culture from those wells with no visible growth was plated on Luria Broth agar and incubated at 37 °C for 24 h. Ampicillin, at different dilutions, was used as a positive control.

### 4.9. Scanning Electron Microscopy

Scanning electron microscopy (SEM) was used to assess the morphological effects of peptides on the *E. coli* and *S. aureus* strains. Both untreated and treated bacteria were fixed overnight at 4 °C with 2.5% glutaraldehyde in 0.1 M sodium cacodylate buffer (pH of 7.4) and post-fixed with 1% osmium tetroxide. After washing with cacodylate buffer, the samples were seeded onto glass slides coated with ε-poly-L-lysine (Sigma-Aldrich, Saint Louis, MO, USA). Adsorbed bacteria were dehydrated using an alcohol gradient (35 to 100%) followed by treatment with hexamethyldisilazane (Sigma–Aldrich, MO, USA) and air drying. The dried specimens were mounted on stubs containing adhesives and sputter-coated with gold. Morphological analysis was performed using an ultra-high-resolution Field Emission Gun Scanning Electron Microscope (FEG-SEM, FEI, Hillsboro, OR, USA). Secondary electron images were taken with an acceleration voltage of 20 kV. The images were processed for display using Photoshop CS4 (version 11.0) software (Adobe Systems Inc., San Jose, CA, USA).

## Figures and Tables

**Table 1:** Main features of the 11 putative antimicrobials salivary candidates selected for this study. Gene names and AGAP codes are reported in the first two columns. Transcriptional profile indicates expression (i) enriched in or specific to female glands (SG+), (ii) restricted to female and male glands (SG F/M), and (iii) ubiquitous (U), i.e., expressed in salivary glands of both sexes and in other tissues. AA, length in amino acids number; kDa, molecular weight; SP, predicted signal peptide length; pI, isoelectric point. In bold, peptides with amino acids length < 60 aa.

Genes	AGAP ID	Transcriptional Profile			Precursor		Putative Propeptide				Putative Mature		
		SG+	SG F/M	U	AA	kDa	SP	AA	kDa	pI	AA	kDa	pI
*hyp13*	AGAP003474			X	56	6.2	22	**34**	3.8	4.75	**19**	2.0	3.77
*hyp15*	AGAP000152	X			78	9.9	30	**48**	4.9	10.55			
*hyp6.2*	AGAP006495	X			85	9.3	27	**58**	6.3	10.41	**35**	4.0	11.57
*hyp6.3*	AGAP007195		X		83	8.8	21	62	6.5	6.29			
*hyp10*	AGAP008307		X		90	10.0	19	67	7.5	5.42	63	7.0	5.77
*hyp12*	AGAP008306		X		92	10.0	21	71	7.9	4.47			
*hyp8.2*	AGAP006494	X			91	9.8	18	73	7.9	4.19			
*sg2*	AGAP006506		X		114	11.8	20	94	9.7	3.49			
*Ag_sal_Lyzo1*	AGAP007347			X	140	15.3	20	120	13.3	8.91			
*hyp14.5*	AGAP004883			X	180	19.7	26	154	16.8	8.07			
*gSG9*	AGAP013423		X		393	42.7	23	370	40.1	5.78	148	15.6	5.76
*hyp55.3*	AGAP005822		X		513	55.2	21	492	52.9	8.73	276	29.8	8.77

**Table 2:** Primers used for the RT-PCR amplification of the selected genes as indicated. NcoI and SmaI restriction sites were inserted at the 5′ end of forward and reverse primers, respectively. Forward primers include an ATG start codon within the NcoI cloning site and allow for the transcription of recombinant peptides from the first amino acid residue of the mature peptide. Reverse primers allow for the addition of a 6-histidine tag upstream of the stop codon, TAA, present in the pIVEX 1.3 plasmid.

Gene	AGAP ID	ID pr. For	Primer for Sequence	ID pr. Rev	Primer Rev Sequence	Length
*hyp13*	AGAP003474	03474_F	GTCACCATGGGGAACGAAATCATACAAAA	03474_R	GTCACCCGGGTTGCGATCCGGAGTCACTGT	105
*hyp15*	AGAP000152	00152_F	GTCACCATGGATCCACTGCCGGGCAGAGA	00152_R	GTCACCCGGGCATGTTTGTTAATACACCGC	147
*hyp6.2*	AGAP006495	06495_F	GTCACCATGGCTCCACAAGTGACTGAGGC	06495_R	GTCACCCGGGCTTTTTCACTCGCAAAAAAT	177
*hyp6.3*	AGAP007195	07195_F	GTCACCATGGTGCCTCAACCTGAGCAGGCC	07195_R	GTCACCCGGGGCAATCAATCAGATCGCAAC	189
*hyp10*	AGAP008307	08307_F	GTCACCATGGAAGACCCCCGTACCGAGCT	08307_R	GTCACCCGGGGCGAATATCCTTTGTACAGT	204
*hyp12*	AGAP008306	08306_F	GTCACCATGGGAAACGATCCAGTCGATGCACT	08306_R	GTCACCCGGGTTGTATATTCTTAGTACAGT	216
*hyp8.2*	AGAP006494	06494_F	GTCACCATGGAAGAAGCTAGTACCGCAGC	06494_R	GTCACCCGGGGCCTGAAAACGAGAAGGGCA	222
*sg2*	AGAP006506	06506_F	GTCACCATGGTTCCGACCAGCTTCAACTAC	06506_R	GTCACCCGGGTCCGAAGAACGGAAAACCTC	285
*Ag_sal_Lyzo1*	AGAP007347	07347_F	GTCACCATGGGTAAAACGTTCGGCAAATGTG	07347_R	GTCACCCGGGAAAACAGGAGCTAACATTCG	363
*hyp14.5*	AGAP004883	04883_F	GTCACCATGGTGCAATGTCGCAACTGTCTA	04883_R	GTCACCCGGGTCGTGCTGCTAGAAGAAGAA	465
*gSG9*	AGAP013423	13423_F	GTCACCATGGGTAGTCCATTCTTTTTCCAATA	13423_R	GTCACCCGGGCGAACCAAATACTTGACAAA	1113
*hyp55.3*	AGAP005822	05822_F	GTCACCATGGTGCCCGACTTTGCGGTACCG	05822_R	GTCACCCGGGACTCAAACAGTTGGCAATCG	1479

## Data Availability

The original contributions presented in the study are included in the article/Supplementary Materials, further inquiries can be directed to the corresponding author.

## Supplementary Materials

The following supporting information can be downloaded at https://www.mdpi.com/article/10.3390/ijms25105529/s1.

## References

[1] Ribeiro J.M.C., Mans B.J., Arcà B. (2010). An Insight into the Sialome of Blood-Feeding Nematocera. Insect Biochem. Mol. Biol..

[2] Arcà B., Ribeiro J.M. (2018). Saliva of Hematophagous Insects: A Multifaceted Toolkit. Curr. Opin. Insect Sci..

[3] Vogt M.B., Lahon A., Arya R.P., Kneubehl A.R., Spencer Clinton J.L., Paust S., Rico-Hesse R. (2018). Mosquito Saliva Alone Has Profound Effects on the Human Immune System. PLoS Negl. Trop. Dis..

[4] Schneider C.A., Calvo E., Peterson K.E. (2021). Arboviruses: How Saliva Impacts the Journey from Vector to Host. Int. J. Mol. Sci..

[5] Graumans W., Jacobs E., Bousema T., Sinnis P. (2020). When Is a Plasmodium-Infected Mosquito an Infectious Mosquito?. Trends Parasitol..

[6] Lefteri D.A., Bryden S.R., Pingen M., Terry S., McCafferty A., Beswick E.F., Georgiev G., Van der Laan M., Mastrullo V., Campagnolo P. (2022). Mosquito Saliva Enhances Virus Infection through Sialokinin-Dependent Vascular Leakage. Proc. Natl. Acad. Sci. USA.

[7] Guerrero D., Cantaert T., Missé D. (2020). Aedes Mosquito Salivary Components and Their Effect on the Immune Response to Arboviruses. Front. Cell Infect. Microbiol..

[8] Arora G., Chuang Y.M., Sinnis P., Dimopoulos G., Fikrig E. (2023). Malaria: Influence of Anopheles Mosquito Saliva on Plasmodium Infection. Trends Immunol..

[9] Marín-López A., Raduwan H., Chen T.Y., Utrilla-Trigo S., Wolfhard D.P., Fikrig E. (2023). Mosquito Salivary Proteins and Arbovirus Infection: From Viral Enhancers to Potential Targets for Vaccines. Pathogens.

[10] Accoti A., Damiani C., Nunzi E., Cappelli A., Iacomelli G., Monacchia G., Turco A., D’Alò F., Peirce M.J., Favia G. (2023). Anopheline Mosquito Saliva Contains Bacteria That Are Transferred to a Mammalian Host through Blood Feeding. Front. Microbiol..

[11] Arcà B., Lombardo F., Francischetti I.M.B., Pham V.M., Mestres-Simon M., Andersen J.F., Ribeiro J.M.C., Arcà B., Lombardo F., Francischetti I.M.B. (2007). An Insight into the Sialome of the Adult Female Mosquito Aedes Albopictus. Insect Biochem. Mol. Biol..

[12] Ribeiro J.M.C., Martin-Martin I., Arcà B., Calvo E. (2016). A Deep Insight into the Sialome of Male and Female *Aedes aegypti* Mosquitoes. PLoS ONE.

[13] Arcà B., Lombardo F., Valenzuela J.G., Francischetti I.M.B., Marinotti O., Coluzzi M., Ribeiro J.M.C., Arcà B., Lombardo F., Valenzuela J.G. (2005). An Updated Catalogue of Salivary Gland Transcripts in the Adult Female Mosquito, Anopheles Gambiae. J. Exp. Biol..

[14] Luo E., Matsuoka H., Yoshida S., Iwai K., Arai M., Ishii A. (2000). Changes in Salivary Proteins during Feeding and Detection of Salivary Proteins in the Midgut after Feeding in a Malaria Vector Mosquito, Anopheles Stephensi (Diptera: Culicidae). Med. Entomol. Zool..

[15] Pascini T.V., Jeong Y.J., Huang W., Pala Z.R., Sá J.M., Wells M.B., Kizito C., Sweeney B., Alves e Silva T.L., Andrew D.J. (2022). Transgenic Anopheles Mosquitoes Expressing Human PAI-1 Impair Malaria Transmission. Nat. Commun..

[16] Klug D., Blandin S.A. (2023). Activation of Complement-like Antiparasitic Responses in Anopheles Mosquitoes. Curr. Opin. Microbiol..

[17] Ribeiro J.M.C., Arca B., Lombardo F., Calvo E., Phan V.M., Chandra P.K., Wikel S.K., Arcà B., Lombardo F., Calvo E. (2007). An Annotated Catalogue of Salivary Gland Transcripts in the Adult Female Mosquito, *Aedes aegypti*. BMC Genom..

[18] Ribeiro J., Arcà B. (2009). From Sialomes to the Sialoverse: An Insight into Salivary Potion of Blood-Feeding Insects. Adv. Insect Phys..

[19] Fry B., Roelants K. (2009). The Toxicogenomic Multiverse: Convergent Recruitment of Proteins into Animal Venoms. Annu. Rev. Genom. Hum. Genet..

[20] James A.A., Rossignol P.A. (1991). Mosquito Salivary Glands: Parasitological and Molecular Aspects. Parasitol. Today.

[21] Rossignol P.A., Lueders A.M. (1986). Bacteriolytic Factor in the Salivary Glands of *Aedes aegypti*. Comp. Biochem. Physiol. B.

[22] Calvo E., Pham V.M., Lombardo F., Arca B., Ribeiro J.M. (2006). The Sialotranscriptome of Adult Male Anopheles Gambiae Mosquitoes. Insect Biochem. Mol. Biol..

[23] Rosinski-Chupin I., Briolay J., Brouilly P., Perrot S., Gomez S.M., Chertemps T., Roth C.W., Keime C., Gandrillon O., Couble P. (2007). SAGE Analysis of Mosquito Salivary Gland Transcriptomes during Plasmodium Invasion. Cell Microbiol..

[24] Rosinski-Chupin I., Chertemps T., Boisson B., Perrot S., Bischoff E., Briolay J., Couble P., Ménard R., Brey P., Baldacci P. (2007). Serial Analysis of Gene Expression in *Plasmodium berghei* Salivary Gland Sporozoites. BMC Genom..

[25] Pinheiro-Silva R., Borges L., Coelho L.P., Cabezas-Cruz A., Valdés J.J., do Rosário V., de la Fuente J., Domingos A. (2015). Gene Expression Changes in the Salivary Glands of Anopheles Coluzzii Elicited by *Plasmodium berghei* Infection. Parasit. Vectors.

[26] Chowdhury A., Modahl C.M., Tan S.T., Wei Xiang B.W., Missé D., Vial T., Kini R.M., Pompon J.F. (2020). JNK Pathway Restricts DENV2, ZIKV and CHIKV Infection by Activating Complement and Apoptosis in Mosquito Salivary Glands. PLoS Pathog..

[27] Bevivino G., Arcà B., Lombardo F. (2021). Effects of Local and Systemic Immune Challenges on the Expression of Selected Salivary Genes in the Malaria Mosquito Anopheles Coluzzii. Pathogens.

[28] Das De T., Sharma P., Thomas T., Singla D., Tevatiya S., Kumari S., Chauhan C., Rani J., Srivastava V., Kaur R. (2018). Interorgan Molecular Communication Strategies of “Local” and “Systemic” Innate Immune Responses in Mosquito Anopheles Stephensi. Front. Immunol..

[29] Clayton A.M., Dong Y., Dimopoulos G. (2014). The Anopheles Innate Immune System in the Defense against Malaria Infection. J. Innate Immun..

[30] Osta M.A., Christophides G.K., Kafatos F.C. (2004). Effects of Mosquito Genes on Plasmodium Development. Science.

[31] Saraiva R.G., Kang S., Simões M.L., Angleró-Rodríguez Y.I., Dimopoulos G. (2016). Mosquito Gut Antiparasitic and Antiviral Immunity. Dev. Comp. Immunol..

[32] Tikhe C.V., Dimopoulos G. (2021). Mosquito Antiviral Immune Pathways. Dev. Comp. Immunol..

[33] Baltzer S.A., Brown M.H. (2011). Antimicrobial Peptides-Promising Alternatives to Conventional Antibiotics. J. Mol. Microbiol. Biotechnol..

[34] Manniello M.D., Moretta A., Salvia R., Scieuzo C., Lucchetti D., Vogel H., Sgambato A., Falabella P. (2021). Insect Antimicrobial Peptides: Potential Weapons to Counteract the Antibiotic Resistance. Cell. Mol. Life Sci..

[35] Hancock R.E.W., Sahl H.G. (2006). Antimicrobial and Host-Defense Peptides as New Anti-Infective Therapeutic Strategies. Nat. Biotechnol..

[36] Tonk M., Vilcinskas A. (2017). The Medical Potential of Antimicrobial Peptides from Insects. Curr. Top. Med. Chem..

[37] Jin G., Weinberg A. (2019). Human Antimicrobial Peptides and Cancer. Semin. Cell Dev. Biol..

[38] Stuart B.A.R., Franitza A.L., E L. (2022). Regulatory Roles of Antimicrobial Peptides in the Nervous System: Implications for Neuronal Aging. Front. Cell Neurosci..

[39] Yi H.Y., Chowdhury M., Huang Y.D., Yu X.Q. (2014). Insect Antimicrobial Peptides and Their Applications. Appl. Microbiol. Biotechnol..

[40] Mahlapuu M., Björn C., Ekblom J. (2020). Antimicrobial Peptides as Therapeutic Agents: Opportunities and Challenges. Crit. Rev. Biotechnol..

[41] Lai Y., Gallo R.L. (2009). AMPed up Immunity: How Antimicrobial Peptides Have Multiple Roles in Immune Defense. Trends Immunol..

[42] Kumar A., Srivastava P., Sirisena P.D.N.N., Dubey S.K., Kumar R., Shrinet J., Sunil S. (2018). Mosquito Innate Immunity. Insects.

[43] Zhang R., Zhu Y., Pang X., Xiao X., Zhang R., Cheng G. (2017). Regulation of Antimicrobial Peptides in *Aedes aegypti* Aag2 Cells. Front. Cell Infect. Microbiol..

[44] Gendrin M., Christophides G.K. (2013). The Anopheles Mosquito Microbiota and Their Impact on Pathogen Transmission. Anopheles Mosquitoes—New Insights into Malaria Vectors.

[45] Hultmark D., Steiner H., Rasmuson T., Boman H.G. (1980). Insect Immunity. Purification and Properties of Three Inducible Bactericidal Proteins from Hemolymph of Immunized Pupae of Hyalophora Cecropia. Eur. J. Biochem..

[46] Steiner H., Hultmark D., Engström Å., Bennich H., Boman H.G. (1981). Sequence and Specificity of Two Antibacterial Proteins Involved in Insect Immunity. Nature.

[47] Wang X., Wang G. (2016). Insights into Antimicrobial Peptides from Spiders and Scorpions. Protein Pept. Lett..

[48] Mylonakis E., Podsiadlowski L., Muhammed M., Vilcinskas A. (2016). Diversity, Evolution and Medical Applications of Insect Antimicrobial Peptides. Philos. Trans. R. Soc. B Biol. Sci..

[49] Erdem Büyükkiraz M., Kesmen Z. (2022). Antimicrobial Peptides (AMPs): A Promising Class of Antimicrobial Compounds. J. Appl. Microbiol..

[50] Lazzaro B.P., Zasloff M., Rolff J. (2020). Antimicrobial Peptides: Application Informed by Evolution. Science.

[51] Rima M., Rima M., Fajloun Z., Sabatier J.M., Bechinger B., Naas T. (2021). Antimicrobial Peptides: A Potent Alternative to Antibiotics. Antibiotics.

[52] Arcà B., Lombardo F., Struchiner C.J., Ribeiro J.M.C. (2017). Anopheline Salivary Protein Genes and Gene Families: An Evolutionary Overview after the Whole Genome Sequence of Sixteen Anopheles Species. BMC Genom..

[53] Moreira-Ferro C.K., Daffre S., James A.A., Marinotti O. (1998). A Lysozyme in the Salivary Glands of the Malaria Vector Anopheles Darlingi. Insect Mol. Biol..

[54] Wong E.S.W., Hardy M.C., Wood D., Bailey T., King G.F. (2013). SVM-Based Prediction of Propeptide Cleavage Sites in Spider Toxins Identifies Toxin Innovation in an Australian Tarantula. PLoS ONE.

[55] Pineda S.S., Chaumeil P.A., Kunert A., Kaas Q., Thang M.W.C., Le L., Nuhn M., Herzig V., Saez N.J., Cristofori-Armstrong B. (2018). ArachnoServer 3.0: An Online Resource for Automated Discovery, Analysis and Annotation of Spider Toxins. Bioinformatics.

[56] Duckert P., Brunak S., Blom N. (2004). Prediction of Proprotein Convertase Cleavage Sites. Protein Eng. Des. Sel..

[57] Maharaj P.D., Widen S.G., Huang J., Wood T.G., Thangamani S. (2015). Discovery of Mosquito Saliva MicroRNAs during CHIKV Infection. PLoS Negl. Trop. Dis..

[58] Arcà B., Colantoni A., Fiorillo C., Severini F., Benes V., Di Luca M., Calogero R.A., Lombardo F. (2019). MicroRNAs from Saliva of Anopheline Mosquitoes Mimic Human Endogenous MiRNAs and May Contribute to Vector-Host-Pathogen Interactions. Sci. Rep..

[59] Fiorillo C., Yen P.S., Colantoni A., Mariconti M., Azevedo N., Lombardo F., Failloux A.B., Arcà B. (2022). MicroRNAs and Other Small RNAs in *Aedes aegypti* Saliva and Salivary Glands Following Chikungunya Virus Infection. Sci. Rep..

[60] Ribeiro J.M. (1987). Vector Salivation and Parasite Transmission. Mem. Inst. Oswaldo Cruz.

[61] Pimenta P.F., Touray M., Miller L. (1994). The Journey of Malaria Sporozoites in the Mosquito Salivary Gland. J. Eukaryot. Microbiol..

[62] Ghosh A.K., Jacobs-Lorena M. (2009). Plasmodium Sporozoite Invasion of the Mosquito Salivary Gland. Curr. Opin. Microbiol..

[63] Klug D., Gautier A., Calvo E., Marois E., Blandin S.A. (2023). The Salivary Protein Saglin Facilitates Efficient Midgut Colonization of *Anopheles* Mosquitoes by Malaria Parasites. PLoS Pathog..

[64] Chaerkady R., Kelkar D.S., Muthusamy B., Kandasamy K., Dwivedi S.B., Sahasrabuddhe N.A., Kim M.S., Renuse S., Pinto S.M., Sharma R. (2011). A Proteogenomic Analysis of Anopheles Gambiae Using High-Resolution Fourier Transform Mass Spectrometry. Genome Res..

[65] Vlachou D., Schlegelmilch T., Christophides G.K., Kafatos F.C. (2005). Functional Genomic Analysis of Midgut Epithelial Responses in Anopheles during Plasmodium Invasion. Curr. Biol..

[66] Byrd A.L., Belkaid Y., Segre J.A. (2018). The Human Skin Microbiome. Nat. Rev. Microbiol..

[67] Burian M., Plange J., Schmitt L., Kaschke A., Marquardt Y., Huth L., Baron J.M., Hornef M.W., Wolz C., Yazdi A.S. (2021). Adaptation of Staphylococcus Aureus to the Human Skin Environment Identified Using an Ex Vivo Tissue Model. Front. Microbiol..

[68] Blount Z.D. (2015). The Unexhausted Potential of *E. coli*. eLife.

[69] Lombardo F., Ronca R., Rizzo C., Mestres-Simon M., Lanfrancotti A., Curra C., Fiorentino G., Bourgouin C., Ribeiro J.M.C., Petrarca V. (2009). The Anopheles Gambiae Salivary Protein GSG6: An Anopheline-Specific Protein with a Blood-Feeding Role. Insect Biochem. Mol. Biol..

[70] Francischetti I. (2002). Toward a Catalog for the Transcripts and Proteins (Sialome) from the Salivary Gland of the Malaria Vector Anopheles Gambiae. J. Exp. Biol..

[71] Calvo E., Mans B.J., Andersen J.F., Ribeiro J.M. (2006). Function and Evolution of a Mosquito Salivary Protein Family. J. Biol. Chem..

[72] Marinotti O., James A.A., Ribeiro J. (1990). Diet and Salivation in Female *Aedes aegypti* Mosquitoes. J. Insect Physiol..

[73] Novak M.G., Ribeiro J.M.C., Hildebrand J.G. (1995). 5-Hydroxytryptamine in the Salivary Glands of Adult Female *Aedes aegypti* and Its Role in Regulation of Salivation. J. Exp. Biol..

[74] Li Z., Soohoo-Hui A., O’Hara F.M., Swale D.R. (2022). ATP-Sensitive Inward Rectifier Potassium Channels Reveal Functional Linkage between Salivary Gland Function and Blood Feeding in the Mosquito, *Aedes aegypti*. Commun. Biol..

[75] Gómez-Díaz E., Rivero A., Chandre F., Corces V.G. (2014). Insights into the Epigenomic Landscape of the Human Malaria Vector Anopheles Gambiae. Front. Genet..

[76] Ruiz J.L., Ranford-Cartwright L.C., Gómez-Díaz E. (2021). The Regulatory Genome of the Malaria Vector Anopheles Gambiae: Integrating Chromatin Accessibility and Gene Expression. NAR Genom. Bioinform..

[77] Jeffery G.M. (1956). Blood Meal Volume in Anopheles Quadrimaculatus, A. Albimanus and *Aedes aegypti*. Exp. Parasitol..

[78] Graumans W., Heutink R., Van Gemert G.J., Van De Vegte-Bolmer M., Bousema T., Collins K.A. (2020). A Mosquito Feeding Assay to Examine Plasmodium Transmission to Mosquitoes Using Small Blood Volumes in 3D Printed Nano-Feeders. Parasites Vectors.

[79] Jové V., Gong Z., Hol F.J.H., Zhao Z., Sorrells T.R., Carroll T.S., Prakash M., McBride C.S., Vosshall L.B. (2020). Sensory Discrimination of Blood and Floral Nectar by *Aedes aegypti* Mosquitoes. Neuron.

[80] Hajam I.A., Dar P.A., Won G., Lee J.H. (2017). Bacterial Ghosts as Adjuvants: Mechanisms and Potential. Vet. Res..

[81] Ma Y., Zhu W., Zhu G., Xu Y., Li S., Chen R., Chen L., Wang J. (2022). Efficient Robust Yield Method for Preparing Bacterial Ghosts by Escherichia Coli Phage ID52 Lysis Protein E. Bioengineering.

[82] Chen R.-B., Zhang K., Zhang H., Gao C.-Y., Li C.-L. (2018). Analysis of the Antimicrobial Mechanism of Porcine Beta Defensin 2 against *E. coli* by Electron Microscopy and Differentially Expressed Genes. Sci. Rep..

[83] Huan Y., Kong Q., Mou H., Yi H. (2020). Antimicrobial Peptides: Classification, Design, Application and Research Progress in Multiple Fields. Front. Microbiol..

[84] Roversi D., Luca V., Aureli S., Park Y., Mangoni M.L., Stella L. (2014). How Many Antimicrobial Peptide Molecules Kill a Bacterium? The Case of PMAP-23. ACS Chem. Biol..

[85] Chongsiriwatana N.P., Lin J.S., Kapoor R., Wetzler M., Rea J.A.C., Didwania M.K., Contag C.H., Barron A.E. (2017). Intracellular Biomass Flocculation as a Key Mechanism of Rapid Bacterial Killing by Cationic, Amphipathic Antimicrobial Peptides and Peptoids. Sci. Rep..

[86] Varadi M., Anyango S., Deshpande M., Nair S., Natassia C., Yordanova G., Yuan D., Stroe O., Wood G., Laydon A. (2022). AlphaFold Protein Structure Database: Massively Expanding the Structural Coverage of Protein-Sequence Space with High-Accuracy Models. Nucleic Acids Res..

