# Supplementary material for: Peptides with Antimicrobial Activity in the Saliva of the Malaria Vector Anopheles coluzzii
Source: Int J Mol Sci. 2024 May 18;25(10):5529. doi: 10.3390/ijms25105529 (PMC11121840; doi:10.3390/ijms25105529)
Supplement: Supplementary file 1 [file ijms-25-05529-s001.zip › ijms-3008774-supplementary.pdf]

Expression of recombinant peptides using the WG system

Candidate recombinant peptides were obtained employing the cell-free *in vitro* transcription-translation system based on wheat germ extract and a continuous exchange cell-free reaction system for protein synthesis (RTS^{TM} 100 Wheat Germ CECF Kit - 5PRIME). For each candidate (Table S1), primers were designed to allow PCR amplification and subsequent cloning in the expression vector pIVEX-1.3-His-tagged.

Table S1. Candidates selected for wheat germ cell-free *in vitro* transcription-translation system.

		Transcriptional profile			Full		Precursor				Mature		
Genes	AGAP ID	SG+	SG F/M	U	AA	kDa	SP	*AA*	kDa	pI	AA	kDa	pI
hyp6.3 putative secreted salivary protein	AGAP007195		X		83	8.8	21	*62*	6.5	6.29			
hyp10 hypothetical salivary protein 10	AGAP008307		X		90	10.0	19	*67*	7.5	5.42	63	7.0	5.77
hyp12 hypothetical salivary protein 12	AGAP008306		X		92	10.0	21	*71*	7.9	4.47			
hyp8.2 hypothetical salivary protein 8.2	AGAP006494	X			91	9.8	18	*73*	7.9	4.19			
sg2 salivary protein	AGAP006506		X		114	11.8	20	*94*	9.7	3.49			
Ag_sal_Lyzo1 abundant salivary lysozyme	AGAP007347			X	140	15.3	20	*120*	13.3	8.91			
hyp14.5 similar to Culex 14.5 kDa sal. pep.	AGAP004883			X	180	19.7	26	*154*	16.8	8.07			
gSG9 protein	AGAP013423		X		393	42.7	23	*370*	40.1	5.78	148	15.6	5.76
hyp55.3 putative 55.3 salivary protein	AGAP005822		X		513	55.2	21	*492*	52.9	8.73	276	29.8	8.77

In vitro transcription/translation led to the production of 9 recombinant peptide (Figure S1).

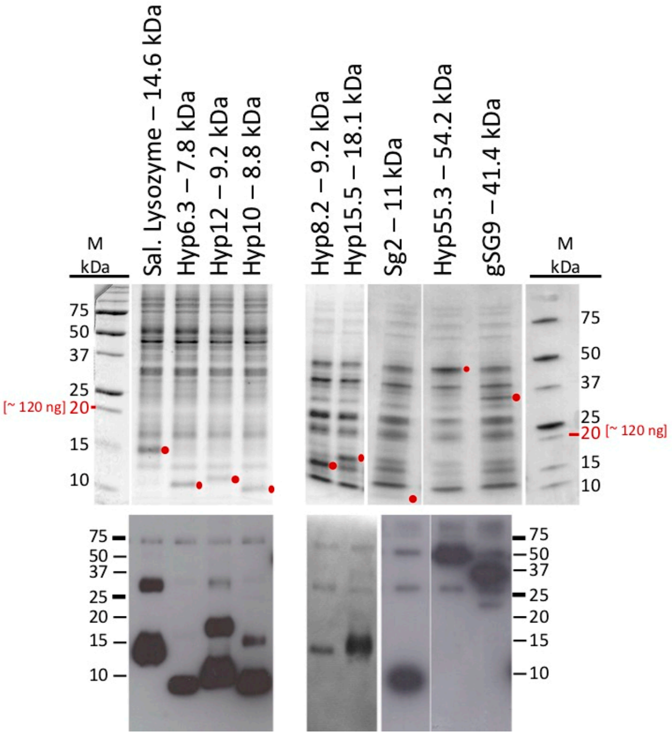

Figure S1. Wheat germ extracts (0.5 µl) including the recombinat pIVEX vector encoding the polypeptide indicated on the top were separated by SDS-PAGE and stained by Coomassie (upper panel) or analyzed by Western blot using an anti-His tag monoclonal antibody (lower panel). Bands

corresponding to the different recombinant proteins are indicated by the red dots. The 20 kDa band from the Molecular Weight Marker was used as reference for estimation of protein yield.

A rough quantitative estimation of the recombinant peptides was obtained from the Coomassie stained acrylamide gels, with yields varying between 10 and 45 micrograms for 50 µl of crude extract (Table S2 and Figure S1).

Table S2. Salivary candidates (Genes and AGAP ID columns) and the corresponding main results of wheat germ cell-free *in vitro* transcription-translation experiments: length and molecular weight of recombinant peptides (Aa Rec. Pr. and kDa columns) and estimated yields of each recombinant peptide (ng/lane, µg/50 µl, µM) are reported.

**Genes**	**AGAP ID**	**Aa Rec. Pr.**	**kDa**	**ng/lane**	**µg/50µl**	**µM**
hyp8.2 hypothetical salivary p. 8.2	AGAP006494	85	9.2	295,91	29.6	~64
hyp55.3 putative 55.3 salivary protein	AGAP005822	504	54.2	445,79	44.6	~16
sg2 salivary protein	AGAP006506	106	11	131,53	13.1	~23
hyp6.3 putative secreted salivary	AGAP007195	74	7.8	142,85	14.3	~36
hyp12 hypothetical salivary protein 12	AGAP008306	83	9.2	98,63	9.9	~22
hyp10 hypothetical salivary protein 10	AGAP008307	79	8.8	107,27	10.7	~24
gSG9 protein	AGAP013423	382	41.4	412,56	41.2	~20
hyp14.5 similar to Culex q. 14.5 kda sal. pep.	AGAP004883	166	18.1	241,59	24.2	~26
Ag_sal_Lyzo1 abundant salivary lysozyme	AGAP007347	132	14.6	287,50	28.7	~39

As also explained in the main text, due to the limited amounts of peptides, it was not possible to proceed with purification of the his-tagged recombinant proteins. Therefore, to try to evaluate anyway the possible antimicrobial activity of the selected candidates, crude extracts containing the recombinant peptides were used in bacterial growth inhibition assays.

Bacterial growth inhibition assays

The assays were conducted in 96-well plates (see Materials and Methods), monitoring the growth of bacteria in the presence of i) wheat germ crude extracts after addition of the empty pIVEX plasmid (negative control); ii) wheat germ crude extracts after addition of recombinant pIVEX plasmids encoding candidate polypeptides (Figure S2).

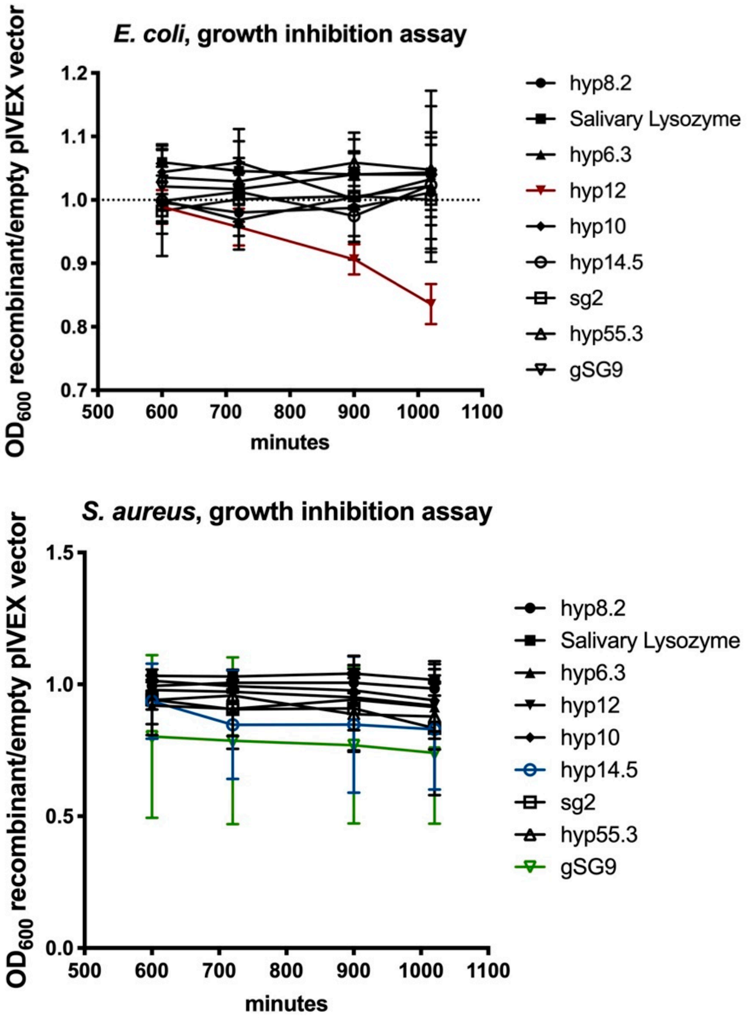

Figure S2. Bacterial growth inhibition assays using crude wheat germ extracts. Bacteria were grown O/N in presence of crude extracts including the recombinant peptides as indicated in the legends. The ratio between OD_{600} of bacterial cultures with and without the recombinant peptides (i.e, crude extracts including recombinant or empty pIVEX) at the different timepoints (from approximately 10 to 15 hours) are shown for *E. coli* ATCC25922 (top) and *S. aureus* ATCC25923 (bottom). Results from biological triplicates are shown.

Main properties of chemically synthesized peptides

Peptides that after removal of the signal peptide were shorter than 60 aminoacids were chemically synthesized. Table S3 reports the main features of hyp6.2, hyp13 and hyp15 with predicted propeptides and mature forms, when applicable.

Table S3. Features of hyp6.2, hyp13 and hyp15. Sequence, molecular weight (DA), length (AA), HPLC purity percent and solvent used for suspension are reported. Mature, short forms of hyp6.2 and hyp13 are indicated as underlined sequences.

Peptide	Sequence	MW (DA)	Lenght (AA)	HPLC purity	Solvent
**Hyp6.2**	APQVTEAPGNVGSTYSPMADIGRLATGATKLFGQFWNTGTRFGTELSRRTFDFLRVKK	6352.15	**58**	90.4%	DMSO
**Hyp6.2**	LATGATKLFGQFWNTGTRFGTELSRRTFDFLRVKK	4051.63	**35**	95.7%	DMSO
**Hyp13**	NEIIQNVVKRSIPLRQLILQHNALDDSDSDSGSQ	3804.16	**34**	99.7%	ULTRAPURE WATER
**Hyp13**	QLILQHNALDDSDSDSGSQ	2043.07	**19**	97.9%	3% AMMONIA WATER
**Hyp15**	DPLPGRDRNTIANKSKDKKASAPKHSLGTGARMALTGGGVLGGVLTNM	4860.56	**48**	>90%	ULTRAPURE WATER

Growth inhibition assays: hyp15

No significant growth inhibition was induced by the hyp15 peptide at a concentration of 150 µM during the initial tests (Figure S3); for this reason, no additional assays were performed with this peptide (see also main text).

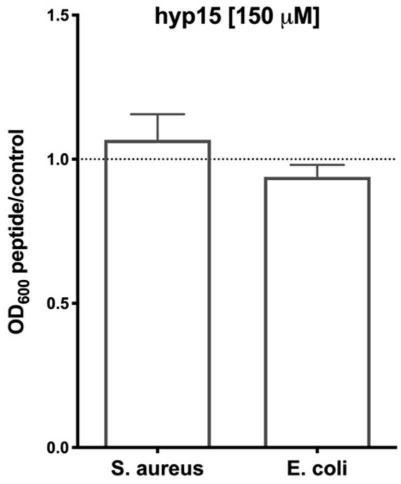

Figure S3. Optical Densities at 600 nm of bacterial cultures after 10 hours of incubation with or without 150 µM of hyp15. The OD_{600} represent the ratio between growth with and without peptide. No significant difference was found by 2-way ANOVA followed by Dunnett's test). Results of at least three replicates are reported.

Growth inhibition assays: hyp6.2 and hyp13

Growth inhibition induced by hyp6.2 or hyp13, both precursors and mature forms, were evaluated by CFU counting (as reported in the main text, Figures 3 and 5) and by measuring the OD of the bacterial culture after O/N incubation with different concentrations of the peptides (as reported in Figure S4).

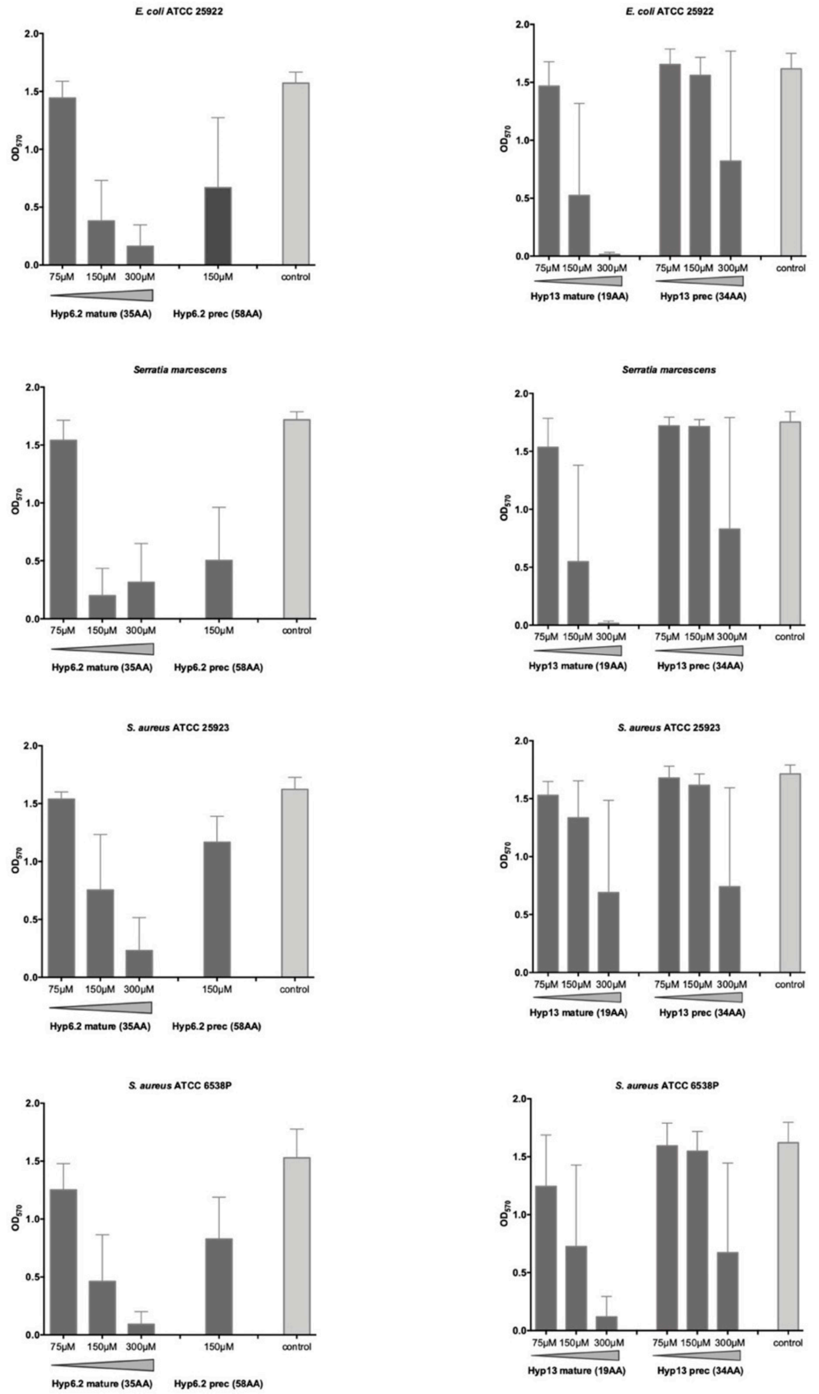

Figure S4. Bacterial growth inhibition assays using the hyp6.2 and hyp13 peptides. The OD_{570} after addition of different concentration of peptides to the different bacterial strains are shown. Average values and standard deviations of three independent experiments are reported.
